# Generative adversarial networks for enhanced performance prediction of square CFST members under axial tension

**DOI:** 10.1371/journal.pone.0349875

**Published:** 2026-06-01

**Authors:** Hongtao Zhang, Yang Liu, Junbo Yan

**Affiliations:** 1 University of Applied Technology, University of Science and Technology Liaoning, Anshan, China; 2 Liaoning Metallurgical Geological 405 Team Co., Ltd., Anshan, Liaoning, China; 3 China 22nd Metallurgical Group Co. Ltd., Metallurgical Engineering Branch, Tangshan, China; China Construction Fourth Engineering Division Corp. Ltd, CHINA

## Abstract

Taking square concrete-filled steel tubular (CFST) members under axial tension as the research object, a three-dimensional mesoscopic finite element model was established based on the experimental results of six specimens. Ten parametric models were further developed to investigate the effects of section size, confinement coefficient, and slenderness ratio on tensile performance. In addition, code-based comparisons and machine learning predictions were carried out. The results indicate that the finite element simulations agree well with the test results, with the ratios of simulation results to test results all being below 0.95, indicating that the simulation predictions are within a reasonable range of the experimental data, which reflects good agreement. The parametric analysis shows that when the confinement coefficient increases from 0 to 0.99, the maximum load rises from 182 kN to 895 kN; when the slenderness ratio increases from 8 to 20, the maximum load exhibits an overall decreasing trend. The code comparison shows that the predictions from the Chinese code are closer to the finite element results, with an average error of approximately 4.57%. To improve prediction accuracy with limited data, a Generative Adversarial Network (GAN)-based data augmentation method was employed. Using both original and WGAN-GP-augmented data, predictive models were developed. Among these models, the Random Forest model achieved the best overall performance. On the augmented test set, the coefficients of determination (R^2^) for ultimate load and displacement prediction reached 0.997 and 0.9855, respectively. The findings provide a reference for tensile performance analysis and rapid assessment of this type of member, demonstrating the effectiveness of GAN-based data augmentation in enhancing predictive accuracy.

## 1 Introduction

The concrete-filled steel tube (CFST) structure combines the advantages of high strength and high ductility of steel with the excellent compressive performance and lower cost of concrete. It has been widely applied in bridge engineering, high-rise and super high-rise buildings, large-span spatial structures, and heavy-load structures [1–3]. Compared with circular CFST, square CFST not only offers better arrangement of components and architectural suitability but also provides easier node connections and structural treatments, which makes it more advantageous in practical engineering applications [4,5]. In particular, under large loads and in complex service environments, square CFST demonstrates excellent overall mechanical performance and adaptability to engineering requirements. Currently, research on CFST worldwide primarily focuses on axial compression, eccentric compression, seismic performance, and compression-bending behaviors. The bearing mechanism, ductility, confinement effect, and design methods of CFST have been systematically understood [6,7].

However, research on the axial tensile performance of CFST is relatively insufficient. Generally, CFST is not designed as a component primarily subjected to tensile forces, but tensile conditions do exist in practical engineering. For example, edge columns of high-rise and super high-rise buildings may experience alternating tension and compression under wind and seismic loads; CFST piles subjected to lateral and impact loads, as well as components in the anchorage zones of bridge cables, truss chord members, and node areas, may also be in axial tension or combined tension-bending states. Therefore, studying the mechanical behavior and load-bearing capacity of square CFST components under axial tension is of significant importance for improving the CFST theoretical system and guiding relevant engineering design [8–10].

Previous studies have indicated that the concrete infill can play a supporting or stiffening role during the tensile process of the steel tube, thereby enhancing the tensile stiffness and bearing capacity of CFST components. Han et al. [11] noted that the steel content is a key factor affecting the axial tensile performance of CFST, and the concrete provides a significant support to the steel, which increases the tensile bearing capacity of square-section specimens by approximately 4%–7% within a certain steel content range. M. Zhou et al. [12] found that the tensile stiffness of square CFST is on average 31.8% higher than that of empty steel tubes, which they attributed to the combined effects of the confinement stiffening effect and tensile stiffening effect between the concrete and the steel tube. Yong Ye et al. [13] conducted axial and eccentric tensile tests on stainless steel tube concrete members and found that the core concrete effectively enhances the tensile strength and stiffness of the members, with the tensile members generally exhibiting good ductility. Yu Deng et al. [14] pointed out through bidirectional eccentric tensile tests and theoretical analysis that the presence of core concrete provides internal support to the steel tube and improves the tensile performance of the member, with the proposed ultimate bearing capacity calculation method showing an average error of about 6.1%. Wei Li et al. [15] studied the axial and eccentric tensile behavior of double-layer CFST members and concluded that the infilled concrete provides additional support to the inner and outer steel tubes, but the existing design methods still need improvements in predicting their tensile bearing capacity.

Overall, existing studies have provided an important basis for understanding the tensile behavior of CFST members; however, systematic research on square CFST members under axial tension remains relatively limited. First, the currently available experimental samples are generally small in number and limited in parameter coverage, which is insufficient to comprehensively reveal the coupled effects of key factors such as section size, slenderness ratio, confinement effect, and material strength on the axial tensile behavior of the members. Second, traditional experimental investigations are time-consuming and costly, while finite element analysis, although capable of capturing the mechanical response and damage evolution in detail, is still inefficient for rapid multi-parameter evaluation and engineering-oriented prediction [16,17]. In addition, machine learning methods have shown strong potential in modeling complex nonlinear relationships in structural engineering, yet their application to the prediction of ultimate load and displacement of square CFST members under axial tension remains scarce. In particular, under small-sample conditions, it is still difficult to directly establish highly reliable predictive models. Therefore, introducing a data augmentation strategy is necessary to alleviate the adverse influence of insufficient samples on model training and prediction performance [18,19].

Motivated by the above considerations, this study focuses on square CFST members subjected to axial tension. A finite element model is first established and validated against available experimental results, followed by parametric analysis and comparisons with existing code-based design formulas. To address the challenges of limited data, the WGAN-GP method is employed to augment the small-sample dataset, and four machine learning models—KNN, Decision Tree, XGBoost, and Random Forest—are developed to predict the ultimate load and displacement of the members. Finally, SHAP anaThe novelty of this study lies in the integration of mesoscopic finite element modeling, comprehensive axial tensile parametric analysis, and WGAN-GP-based data augmentation for predictive modeling. While previous studies have focused on either mesoscopic modeling or machine learning, this study bridges these areas by employing WGAN-GP to enhance prediction accuracy with small datasets. The findings not only deepen the understanding of the mechanical behavior and key parameters of square CFST members under axial tension but also provide a data-driven approach for rapid performance prediction and engineering assessment of such members.

The remainder of this paper is organized as follows. Section 2 describes the finite element modeling procedure, parametric analysis scheme, and machine-learning methodology, including WGAN-GP-based data augmentation. Section 3 presents the numerical simulation, code comparison, machine-learning prediction results, and SHAP-based interpretation. Section 4 summarizes the main conclusions of this study.lysis is adopted to interpret the contribution of key input variables.

## 2 Materials and methods

### 2.1 Test scheme

In Ref. [20], a total of six square CFST specimens were fabricated, with the outer steel tube made of Q235 steel and the core concrete using different strength grades of concrete. All specimens had a square cross-section, with a height of L, an outer steel tube side length of Bi, and an outer steel tube thickness of t0. The specific dimensional parameters are listed in Table 1. It should be noted that these six specimens were adopted in the present study as benchmark cases for validating the finite element model, rather than as newly conducted tests. After the numerical model was validated against the experimental results reported in Ref. [20], additional numerical models were further established to perform the parametric analysis. In this way, the experimental specimens provide the basis for model verification, while the extended numerical models are used to investigate the effects of key parameters on the tensile behavior of square CFST members.

**Table 1:** List of specimens.

Specimen number	*L*/mm	*B*_i_/mm	*t*_0_/mm	*Concrete grades*
A2	510	100	2.75	C40
B0	510	120	3.5	–
B1	510	120	3.5	C30
B2	510	120	3.5	C40
B3	510	120	3.5	C50
C2	510	140	4.5	C40

### 2.2 Finite element module method

The core concrete is typically considered a three-phase heterogeneous composite material consisting of aggregates, mortar matrix, and the interfacial transition zone [21,22]. According to the experimental study in reference [20], the coarse aggregate used is crushed stone with a particle size of 5–15 mm. Based on reference [23], this study idealizes the coarse aggregates as spherical particles and employs a two-stage aggregate distribution method [24], where the large aggregate has a particle size of 7–8 mm and the small aggregate has a particle size of 5–6 mm. The aggregate particle size distribution follows the Fuller curve, as shown in equation (1).


$$\begin{array}{c} P\text{(}D<d_{\text{i}}\text{)}=100Pk\sqrt{d_{\text{i}}/D_{max}} \end{array}$$ (1)

In the mesoscopic modeling process, the “grid mapping” method [25] is used to randomly distribute the aggregates within the mortar matrix, with a thin layer of elements set around the perimeter of the aggregate particles to represent the interfacial transition zone, the thickness of which is taken as 0.5 mm [26]. Based on this, the generated mesoscopic concrete model is placed between the inner and outer steel tubes, thus establishing a three-dimensional mesoscopic numerical analysis model for the concrete-filled steel tube.

The mesoscopic components of the concrete are discretized using eight-node hexahedral reduced integration elements, with an average mesh size of 2.5 mm. This mesh size was selected as a compromise between computational efficiency and numerical accuracy, and is consistent with previous mesoscopic modeling studies of concrete materials. In addition, preliminary simulations with finer mesh sizes were conducted, which showed that further mesh refinement led to only minor variations in the predicted load–strain response and failure characteristics. Therefore, an average mesh size of 2.5 mm was adopted in this study to ensure sufficient accuracy while maintaining reasonable computational cost. The mechanical properties of the aggregates are closely related to the origin of the rock, with material parameters varying for aggregates from different sources. In this study, the elastic modulus of the aggregate is taken as 56 GPa. The interfacial transition zone (ITZ), as the transition region between the aggregate and the mortar matrix, is typically considered the weak part of the concrete due to the presence of internal pores. Given that there is no unified value for the ITZ thickness, and to balance computational accuracy and efficiency, the thickness of the ITZ is set to 0.5 mm in this study. Previous research has shown that the mechanical properties of the ITZ, such as its elastic modulus and strength, are reduced compared to the mortar matrix, and typically exhibit a gradient characteristic. Prokopski [27] suggested that the mechanical property reduction factor for this region is approximately 70%. Therefore, the mechanical parameters of the interface layer are taken as 70% of the corresponding parameters of the mortar matrix. The mechanical parameters of the mesoscopic concrete components are shown in Table 2.

**Table 2:** Material parameters of concrete components.

Microcosmic composition of concrete	Elastic modulus/GPa	compressive strength/MPa	*Tensile strength*/MPa
Aggregate	56.0	--	--
Mortar	32.5	49.1	4.0
ITZ	26.0	39.3	3.2

The outer steel tube has corner regions formed by cold bending, meaning its cross-section is not an ideal right-angle square tube, but consists of both the corner and flat regions. Based on this, the material constitutive relationship for the steel tube in this study adopts the cold-formed steel constitutive model recommended by Abdel-Rahman and Sivakumaran [23]. The nominal stress ($\sigma_{s}$) – nominal strain ($\epsilon_{s}$) relationship for the flat region of the cold-formed steel tube is as follows:


$$\begin{array}{c} \sigma_{s}=\left\{ \begin{array}{c} @c@E_{s}\cdot \epsilon_{s}\text{ (}\epsilon_{s}\leq \epsilon_{e})\\ f_{p}+E_{s1}\cdot \text{(}\epsilon_{s}-\epsilon_{e}\text{) \textasciitilde{}\textasciitilde{}\textasciitilde{}\textasciitilde{}\textasciitilde{}\textasciitilde{}(}\epsilon_{e}\leq \epsilon_{s}\leq \epsilon_{e1})\\ f_{ym}+E_{s2}\cdot \text{(}\epsilon_{s}-\epsilon_{e}\text{) \textasciitilde{}\textasciitilde{}\textasciitilde{}\textasciitilde{}(}\epsilon_{e}\leq \epsilon_{s}\leq \epsilon_{e2})\\ f_{y}+E_{s3}\cdot \text{(}\epsilon_{s}-\epsilon_{e2}\text{) \textasciitilde{}\textasciitilde{}\textasciitilde{}\textasciitilde{}\textasciitilde{}\textasciitilde{}\textasciitilde{}\textasciitilde{}\textasciitilde{}(}\epsilon_{e}\geq \epsilon_{e2}) \end{array}\right. \end{array}$$ (2)

Where $f_{p}=3f_{y}/4$, $f_{ym}=7f_{y}/8$, $\epsilon_{e1}=\epsilon_{e}+f_{y}/\text{(}8E_{s1}\text{)}$, $\epsilon_{e2}=\epsilon_{e1}+f_{y}/\text{(}8E_{s2}\text{)}$, $E_{s1}=E_{s}/2$, $E_{s2}=E_{s}/10$, and $E_{s3}=E_{s}/200$. The calculation formula for the yield strength of the corner region ($f_{y1}$) is as follows:


$$\begin{array}{c} f_{y1}=\left[0.6\times \frac{B_{c}}{\text{(}r/t{\text{)}}^{m}}+0.4\right]f_{y} \end{array}$$ (3)

$$\begin{array}{c} B_{c}=3.69\cdot \text{(}f_{u}/f_{y}\text{)}-0.819\cdot \text{(}f_{u}/f_{y}{\text{)}}^{\text{2}}-1.79 \end{array}$$ (4)

$$\begin{array}{c} m=0.192\cdot \text{(}f_{u}/f_{y}\text{)}-0.068 \end{array}$$ (5)

**Fig 1:**
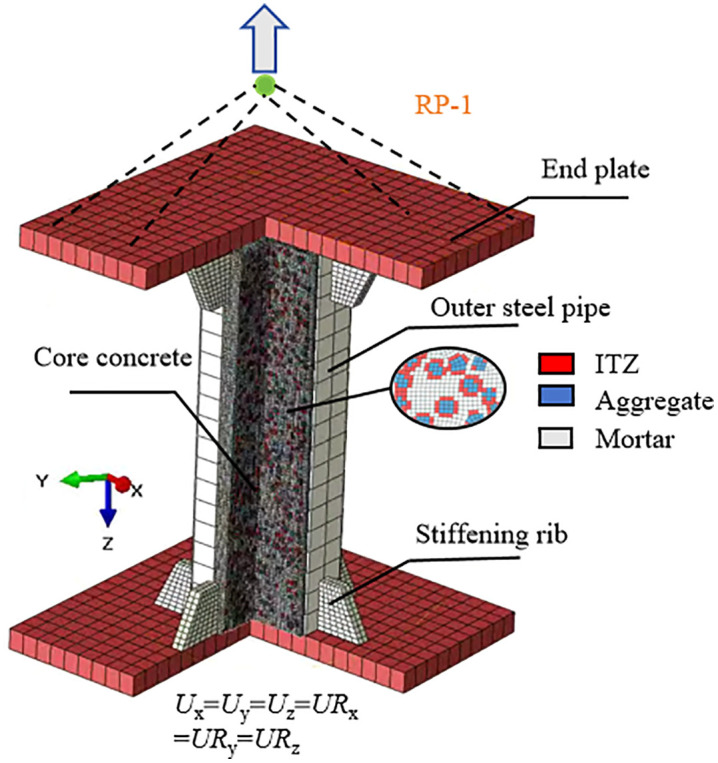
Three-dimensional mesoscopic numerical model of concrete-filled square steel tube.

The normal contact between the interlayer concrete and the steel tube is modeled as “hard” contact, while the tangential contact behavior is represented by interface friction. The critical shear stress is calculated by *τ*_crit_ = μ·p, where *p* is the contact pressure, and the Coulomb friction coefficient *μ* is taken as 0.6 [28,29]. The end plates, outer steel tube, stiffening ribs, and core concrete are all modeled using eight-node reduced integration three-dimensional solid elements (C3D8R). The finite element analysis model after meshing is shown in Fig 1. In the figure, *N* represents the applied axial tensile force (kN), and loading is applied through the rotational displacement in the Z direction. Tie constraints are applied between the end plate, stiffening ribs, and the outer steel tube, and the end plate is set as a rigid body, with its deformation neglected during the loading process. The bottom of the model is fixed, and horizontal translational displacement and rotational degrees of freedom at the loading end are constrained [30,31]. In the contact pair settings, the steel tube surface is defined as the master surface, and the concrete surface is defined as the slave surface.

Based on the experimental validation, this study further establishes finite element models for 10 square concrete-filled steel tube (CFST) columns under axial tension, with specific parameters provided in the relevant parameter table (Table 3). Through parametric analysis, the study focuses on investigating the failure modes and mechanical response patterns, such as size effects, of square CFST columns under different cross-sectional sizes, confinement effect coefficients, and slenderness ratios.

**Table 3:** Parameter analysis model.

Specimen number	L/mm	*B*_i_/mm	*t*_0_/mm	Concrete grades
S0	510	120	0	C40
S1	510	100	2.75	C40
S2	510	120	2.75	C40
S3	510	140	2.75	C40
S4	510	100	3.5	C40
S5	510	120	3.5	C40
S6	510	140	3.5	C40
S7	510	120	4.5	C40
S8	1000	120	3.5	C40
S9	2000	120	3.5	C40

### 2.3 Machine learning model building

On the basis of experimental investigation, finite element simulation, and code-based calculation comparisons, the mechanical behavior and governing factors of this type of member can be systematically revealed. However, finite element modeling is relatively complicated and computationally expensive, while code formulas are generally unable to fully capture the nonlinear response characteristics arising from multi-parameter coupling effects. Therefore, machine learning was further introduced in this study to establish rapid prediction models for the ultimate load and displacement based on the available sample data, thereby providing a supplementary approach for efficient assessment of the mechanical performance of such members [32,33]. The augmented dataset was divided into training and testing sets at a ratio of 8:2 to evaluate the predictive performance of the models within the expanded data domain.

WGAN-GP consists of two components, namely a generator and a discriminator, where the generator is used to produce realistic data samples and the discriminator is employed to assess the authenticity of the generated samples [34,35]. WGAN-GP is an improved version of WGAN, and its main advancement lies in the introduction of a gradient penalty term to replace the weight clipping strategy used in the conventional WGAN. This modification effectively alleviates training instability and better satisfies the Lipschitz continuity condition. Meanwhile, the objective function of the discriminator can be expressed as Eq. (6):


$$\begin{array}{c} L=E_{x\sim P_{r\;}\;}\left[f_{\omega }\;\left(x\right)\right]-E_{x}\sim P_{g}\;\left[f_{\omega }\;\left(x\right)\right] \end{array}$$ (6)

The training hyperparameters of the model are listed in Table 4, where *β*_1_ and *β*_2_ denote the exponential decay rates for the first-order and second-order moment estimates in the Adam optimizer, respectively. Through iterative tuning and optimization of these parameters, the combined effect enables the WGAN-GP model to achieve efficient, stable, and high-quality sample generation when learning complex data distributions. By constraining the Euclidean norm of the gradient of the input samples to remain close to 1, WGAN-GP ensures a more stable training process and improves both the quality and diversity of the generated samples.

**Table 4:** Optimal hyperparameter configuration.

Parameter name	Numerical value
Batch size	64
Number of training rounds/ round	20000
Learning rate	0.0001
Discriminator update frequency	5
Gradient penalty coefficient	8
Adam optimizer *β*_1_	0.9
Adam optimizer *β*_2_	0.001

In this study, the original dataset consists of 16 samples, including 6 experimental specimens and 10 numerical cases. To address the issue of limited data, we employed the WGAN-GP method to augment the dataset. The augmented samples are generated to increase the diversity and quantity of data, helping the machine learning models generalize better. However, it is important to clearly distinguish between real samples (i.e., experimental and numerical data) and generated samples. We carefully validated the generated data to ensure that it closely follows the statistical distributions of the real data, preserving key variable correlations and mechanical response characteristics. Additionally, we evaluated the predictive performance of the models using both the original test set (real samples) and the augmented test set (including generated samples) to confirm that the conclusions drawn from the models are robust.

**Fig 2:**
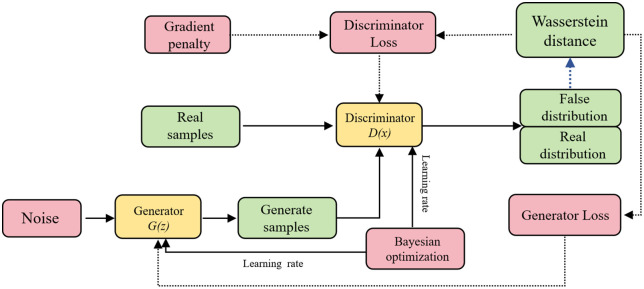
WGAN-GP principle architecture.

In this study, an improved WGAN-GP-based data augmentation method was employed to expand the original dataset, with the aim of enhancing data availability under small-sample conditions. The schematic framework of WGAN-GP is illustrated in Fig 2. The original database consisted of 16 samples in total, including 6 specimens reported in the literature and 10 specimens obtained from numerical simulations [20]. On this basis, WGAN-GP was used to learn the underlying data distribution and generate 100 augmented samples, thereby enlarging the dataset and providing more sufficient data support for the subsequent training of machine learning models.

**Fig 3:**
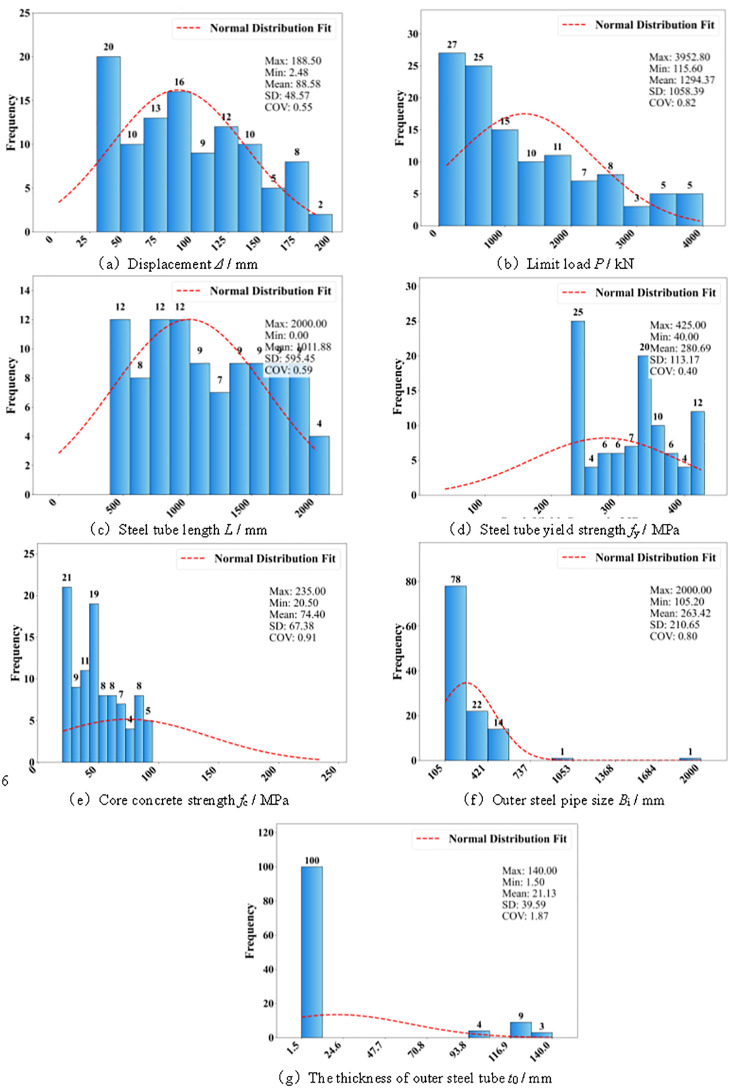
Comparison of characteristic distribution. **(a)** Displacement *Δ*/ mm. **(b)** Limit load *P*/ kN. **(c)** Steel tube length *L*/ mm. **(d)** Steel tube yield strength *f*_y_/ MPa. **(e)** Core concrete strength *f*_c_/ MPa. **(f)** Outer steel pipe size *B*_i_/ mm. **(g)** The thickness of outer steel tube t0/ mm.

Following data augmentation, the outer steel tube size *B*_i_/mm, outer steel tube thickness *t*_0_/mm, steel tube length L/mm, steel tube yield strength *f*_y_/MPa, and core concrete strength *f*_c_/MPa were selected as the input features, while the ultimate load *P*/kN and displacement *Δ*/mm were taken as the output targets to develop the machine learning prediction models. The feature distributions of the augmented dataset are presented in Fig 3. It should be noted that, in the present study, the rationality of the augmented data was only preliminarily validated through feature distribution analysis, whereas the fidelity of the augmented data in preserving variable correlation structures and mechanical response mappings still requires further investigation. In addition, since the testing set was derived from the augmented sample space, the reported results are more focused on reflecting the predictive capability of the models within the expanded data domain, and their generalization performance on independent real samples remains to be further examined in future studies.

For data preprocessing, we applied standardization to ensure that all input features had a mean of 0 and a standard deviation of 1. This was necessary to improve the performance of the machine learning models, especially for algorithms like KNN and XGBoost. Additionally, a fixed random seed was used to ensure reproducibility of the results, and the train-test split was performed once, with the dataset being randomly divided into training and testing subsets.

**Fig 4:**
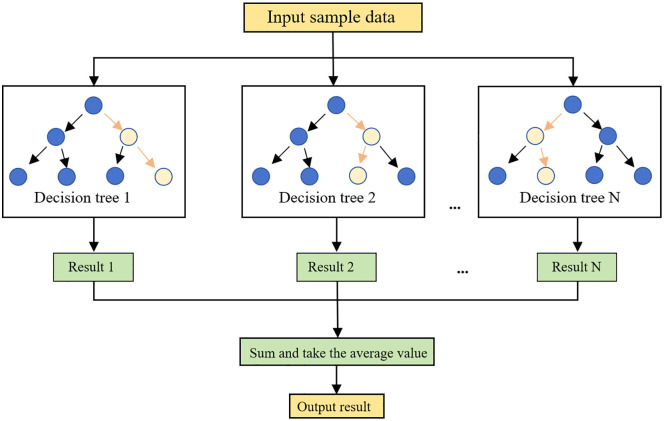
The principle of random forest algorithm.

In terms of predictive modeling, four fundamental machine learning methods, namely KNN [36], Decision Tree [37], XGBoost [38], and Random Forest [39], were employed in this study to predict the ultimate load and displacement, respectively [40–43]. To further illustrate the model construction process, a schematic diagram of the Random Forest model is presented in Fig 4.

## 3 Results

### 3.1 Numerical simulation verification

**Fig 5:**
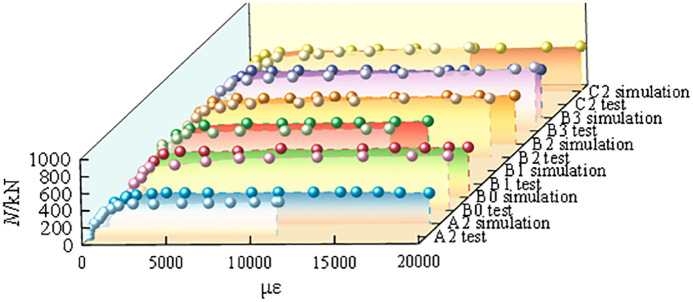
Experimental and numerical simulation verification.

Fig 5 presents a comparison between the experimental results of specimen A2-C2 reported in Ref. [20] and the corresponding numerical simulation results. Overall, the two sets of curves show good agreement, indicating that the established numerical model can reasonably capture the mechanical response characteristics of the specimens considered in this study. In particular, during the initial elastic stage of loading, the slopes of the experimental and simulated curves are nearly identical, suggesting that the mesoscopic model is able to reproduce the stiffness response trend of the specimen with satisfactory accuracy in the elastic range.

It should be noted that some discrepancies still exist between the experimental and numerical results. This is mainly because the core concrete is a three-phase heterogeneous composite material composed of aggregates, mortar matrix, and the interfacial transition zone, whereas the aggregate morphology in the finite element model was idealized. As a result, certain deviations remain between the numerical representation and the actual geometric characteristics of aggregates in real concrete, which in turn affect the predicted ultimate bearing capacity of the specimen. In future studies, aggregate models that more closely resemble the actual morphology could be incorporated into the numerical analysis to further improve simulation accuracy. The numerical model was compared with six experimental specimens, and the results generally show good agreement. To further strengthen the validation, additional quantitative indicators were introduced. The peak load error between the experimental results and the numerical simulations was found to be within 5%, while the initial stiffness error was within 3%. Furthermore, the ratio between the experimental and simulated bearing capacities was consistently less than 0.95, which indicates that the proposed numerical model exhibits good computational consistency within the range of specimens considered in this study. This further supports the reliability of the model for subsequent parametric analyses.

**Fig 6:**
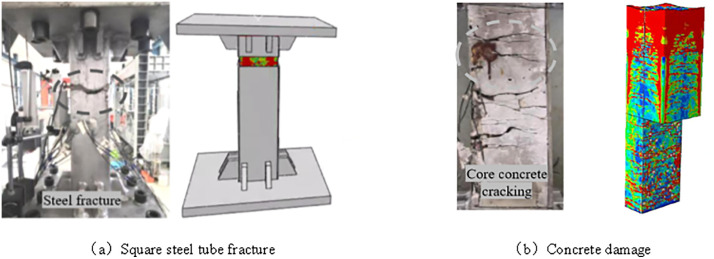
Comparison of test-simulation failure modes. **(a)** Square steel tube fracture. **(b)** Concrete damage.

Specimen B2 is taken as an example for comparison of the failure pattern. Fig 6 shows the comparison between the simulated and experimental failure modes, including the fracture behavior of the outer steel tube (Fig 6(a)) and the damage pattern of the core concrete (Fig 6(b)). As observed in specimen B2, cracking of the steel tube first occurred in the corner region at approximately L/3, and then propagated toward the flat plate region, eventually leading to fracture at the mid-height section. The fracture plane was nearly parallel to the cross-section of the specimen.

After the outer steel tube was cut open, the core concrete was found to remain in a roughly square prismatic shape. Cracks in the internal concrete were symmetrically and relatively uniformly distributed. In the corner region near the mid-span, part of the concrete was crushed, and the crack direction was generally horizontal, with relatively small crack widths at the corners. The cracks primarily propagated from the Interfacial Transition Zone (ITZ) next to the aggregate particles, where the bond between the mortar and the aggregate is weaker. These cracks spread toward the interior of the concrete, following the path of least resistance. Overall, wider cracks were observed in the middle part of the specimen and in regions close to the loading end, whereas the crack widths in other regions were comparatively smaller. This crack pattern and distribution could not be accurately predicted by a homogeneous model, which lacks the ability to account for the localized damage and detailed crack evolution observed in the mesoscopic model.

The three-dimensional mesoscopic numerical simulation shows good agreement with the experimental observations in terms of both the steel tube fracture characteristics (Fig 6(a)) and the concrete damage distribution (Fig 6(b)), indicating that the proposed model can effectively reproduce the typical failure mode of the specimen.

### 3.2 Analysis of the whole process of tensile plastic damage of square steel tube concrete

**Fig 7:**
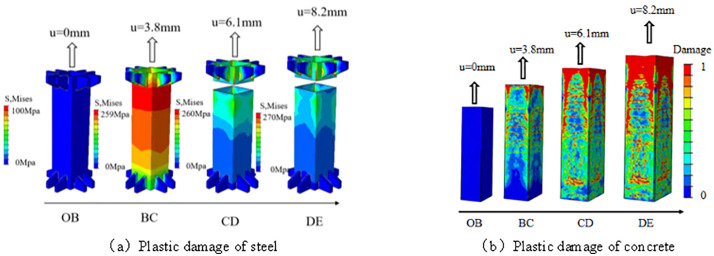
Analysis of the whole process of tensile plastic damage of square steel tube concrete. **(a)** Plastic damage of steel. **(b)** Plastic damage of concrete.

As shown in Fig 7, taking specimen B2 as an example, the mechanical response evolution of the member under axial tension can be divided into four stages. Fig 7(a) illustrates the plastic damage development of the outer steel tube, while Fig 7(b) presents the corresponding plastic damage evolution of the core concrete. During the elastic stage (segment OB), the outer steel tube primarily carries the axial tensile force, with an initial stress of approximately 100 MPa. Owing to the difference in Poisson’s ratio between the steel tube and the core concrete, no pronounced interfacial interaction has yet developed, and the core concrete in Fig 7(b) remains largely intact without obvious cracking.

Upon entering the elastic–plastic stage (segment BC), the local stiffness of the outer steel tube in Fig 7(a) begins to degrade, while the contact and frictional interaction between the steel tube and the concrete gradually intensifies. As a result, the stress distribution starts to become non-uniform, with the stresses in the corner region and the flat plate region reaching about 200 MPa and 150 MPa, respectively. Meanwhile, symmetric transverse cracks begin to appear in the upper part of the core concrete in Fig 7(b), suggesting that under axial tension, the interfacial interaction may already start to influence the local damage evolution of the concrete.

During the elastic–plastic strengthening stage (segment CD), the outer steel tube shown in Fig 7(a) remains the primary load-carrying component. As the tensile deformation continues to develop, the cooperative response between the steel tube and the concrete becomes more evident. Correspondingly, the crack distribution in the upper part of the concrete in Fig 7(b) becomes more uniform, while vertical cracks in the middle region and transverse cracks at the bottom are gradually induced; at this stage, the stress in the corner region increases to approximately 350 MPa. According to the numerical results in this stage, the role of the outer steel tube in the core concrete is manifested more in regulating the crack propagation path and the distribution of local damage, whereas its direct contribution to the tensile response of the concrete remains relatively limited.

When the member enters the plastic stage (segment DE), the outer steel tube in Fig 7(a) gradually yields, with the stresses in the corner region and the flat plate region reaching about 450 MPa and 300 MPa, respectively. At the same time, the concrete damage in Fig 7(b) becomes more pronounced. Under the combined effects of the continuous tensile action in the steel tube and the interfacial interaction, the load–strain curve of the member still exhibits a certain degree of ductile response before final failure.

Overall, under the numerical conditions considered in this study, the outer steel tube shown in Fig 7(a) plays the dominant role in resisting the tensile load, whereas the core concrete in Fig 7(b) participates in the overall structural response mainly through interfacial interaction. As loading proceeds, the redistribution of stress in the steel tube, the enhancement of interfacial friction, and the gradual propagation of cracks in the concrete collectively govern the damage evolution process and the final tensile performance of the member. In contrast to compression members, where the cooperative mechanism is primarily dominated by lateral confinement, the interaction between the steel tube and the concrete in the axial tension members investigated herein is mainly reflected in the suppression of crack propagation, the delay of local damage development, and the improvement of the overall structural working performance.

### 3.3 Parametric analysis

#### 3.3.1 Tensile analysis of concrete filled steel tube under different sections.

**Fig 8:**
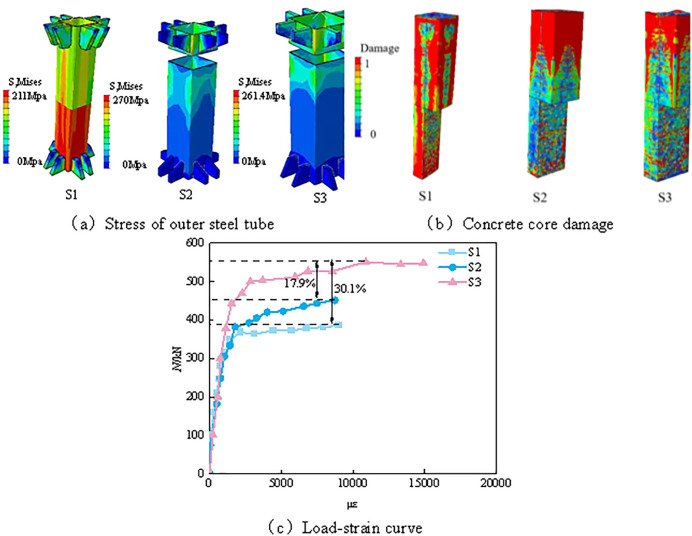
Tensile analysis of concrete filled steel tube under different sections. **(a)** Stress of outer steel tube. **(b)** Concrete core damage. **(c)** Load-strain curve.

As shown in Fig 8(a), in the tensile simulation of concrete-filled steel tubes, the failure pattern of the outer steel tube changes significantly with the increase in the section side length, and the yielding and failure behaviors differ among specimens S1, S2, and S3. For specimen S1, the outer steel tube yields rapidly at the early stage of loading, with the yielding zone mainly concentrated in the middle portion of the tube. The corresponding yield stress is approximately 211 MPa, accompanied by obvious local plastic deformation, indicating a relatively low load-carrying capacity and a higher susceptibility to local yielding failure. As the load continues to increase, the deformation of the outer steel tube gradually intensifies and eventually leads to failure. Compared with S1, specimen S2 exhibits a more uniformly distributed failure pattern of the outer steel tube, with the yielding region extending to the upper part of the tube, indicating a better load-carrying capacity. The failure stress of S2 reaches 270 MPa, which is approximately 28.6% higher than that of S1. The yielding regions in the steel tube are more dispersed, and the strain gradually becomes more uniformly distributed, reflecting improved structural stability. Specimen S3 shows the most uniform failure pattern, with a failure stress of 261.4 MPa, indicating a stronger deformation resistance. Its yielding zone is relatively large, and the overall structure exhibits stronger tensile resistance together with a more stable stress distribution.

As shown in Fig 8(b), with the increase in the specimen section size, the steel ratio gradually decreases, and the confinement efficiency of the steel tube on the core concrete tends to weaken. Correspondingly, the interaction between the steel tube and the core concrete is also reduced to some extent. The tensile cracking of the core concrete gradually shifts from the upper and lower regions toward the upper part of the concrete, accompanied by a large number of transverse cracks in the middle region and a few cracks at the bottom. Under these conditions, the tensile cracks are mainly distributed symmetrically in the transverse direction.

The load–strain curves shown in Fig 8(c) clearly demonstrate the differences in tensile behavior among members with different section sizes. The curve of specimen S1 exhibits a relatively steep ascending trend, indicating that significant strain develops under a relatively low load level and that its load-carrying capacity is limited. The maximum load of S1 is 385.4 kN, corresponding to a maximum microstrain of 9060. At this stage, the steel tube yields rapidly and is unable to effectively carry additional load. In contrast, the curve of specimen S2 is comparatively gentler, indicating a better load-carrying performance. The maximum load of S2 is 451 kN, with a corresponding maximum microstrain of 8731, reflecting a more uniform stress distribution and better stability. As the load increases, the strain develops at a slower rate, suggesting that S2 is able to maintain a relatively stable response under a higher load level. Specimen S3 exhibits the smoothest and most sustained load–strain curve, indicating a stronger tensile resistance. The maximum load of S3 reaches 548 kN, and the corresponding maximum microstrain is 14902. The overall stress distribution is relatively uniform, and its load-carrying capacity is significantly higher than that of S1 and S2. During the loading process, S3 still exhibits a relatively continuous deformation development under high load levels, further demonstrating its superior load-carrying performance.

The above analysis indicates that, as the specimen size increases, the overall structural performance of the CFST member is gradually improved. At the same time, however, the confinement efficiency of the steel tube on the core concrete is weakened, which may affect the overall tensile behavior of the member. It should be noted that the increase in the total bearing capacity resulting from a larger section size is mainly attributed to the increase in geometric dimensions, whereas the decrease in steel ratio leads to a reduction in the confinement efficiency of the steel tube on the core concrete. Therefore, the enhancement in bearing capacity and the weakening of confinement action can coexist.

#### 3.3.2 Analysis of tensile properties of concrete filled steel tube under different constraints.

**Fig 9:**
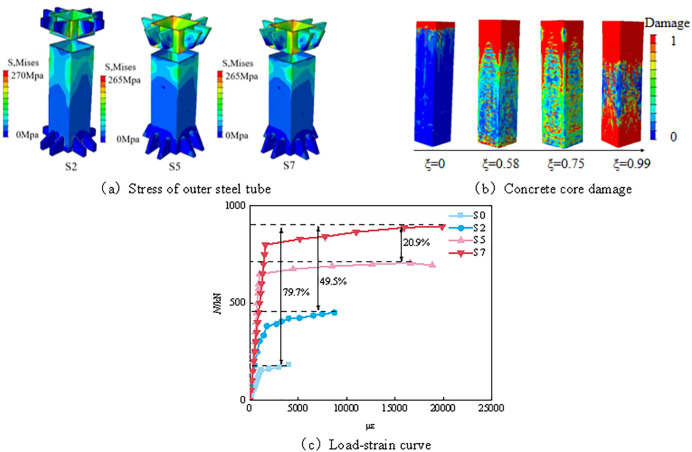
Analysis of tensile properties of concrete filled steel tube under different constraints. **(a)** Stress of outer steel tube. **(b)** Concrete core damage. **(c)** Load-strain curve.


As shown in Fig 9, the confinement effect coefficient can be used to characterize both the compositional and geometric properties of concrete-filled steel tube (CFST) members. It not only reflects the steel content of the composite member, but also, to a large extent, represents the confinement action provided by the steel tube on the core concrete. The expression for the confinement effect coefficient is given as follows:

$$\begin{array}{c} \xi =\frac{A_{\text{s}}f_{\text{y}}}{A_{c}f_{c}} \end{array}$$ (7)

where *A*_s_ is the cross-sectional area of the outer steel tube, *A*_c_ is the void area enclosed within the cross-section of the outer steel tube, *f*_y_ is the yield strength of steel, and *f*_c_ is the axial compressive strength of concrete. Within the scope of the numerical analysis conducted in this study, variations in the confinement effect coefficient have a pronounced influence on the damage pattern and failure mode of the core concrete. The selected numerical models S0, S2, S5, and S7 correspond to confinement effect coefficients of 0, 0.58, 0.75, and 0.99, respectively. The analysis results indicate that, with the increase in the confinement effect coefficient, the damage of the core concrete gradually evolves from localized failure at the upper end to a more uniformly distributed failure pattern.

As shown in Fig 9(b), when the confinement effect coefficient approaches 0, the core concrete mainly exhibits tensile cracking failure at the upper end, while relatively obvious transverse cracks appear at the lower end. Under this condition, due to the absence of confinement and interfacial interaction provided by the outer steel tube, the internal stress of the member is mainly concentrated in the upper part of the concrete, and the damaged region remains relatively localized. As indicated by the load–strain curve in Fig 9(c), the maximum load of model S0 is 182 kN, corresponding to a microstrain of 4000, indicating that this model undergoes pronounced deformation at a relatively low load level and exhibits comparatively weak tensile resistance.

When the confinement effect coefficient increases to 0.58, the damage of the core concrete shown in Fig 9(b) begins to extend from the upper end toward the lower end, and both the upper and lower ends exhibit different degrees of deterioration. As the confinement effect coefficient increases, the interfacial cooperative response between the outer steel tube and the core concrete becomes more pronounced, and the crack propagation path as well as the local damage distribution changes accordingly, resulting in a more coordinated stress state than that under the unconfined condition. Meanwhile, the stress distribution of the outer steel tube in Fig 9(a) also becomes more uniform. The failure stress of model S2 reaches 270 MPa, which is higher than that of S0. As shown in Fig 9(c), its maximum load is 451 kN, with a microstrain of 8731. At this stage, failure is still mainly concentrated in the fracture region of the outer steel tube, indicating that under axial tension, the outer steel tube continues to play the dominant role in resisting the tensile force, whereas the core concrete mainly contributes through its cooperative participation in the overall response.

When the confinement effect coefficient is further increased to 0.75 and 0.99, the damage distribution of the core concrete in Fig 9(b) gradually becomes more uniform, and the failure mode expands from a localized concentration at the upper end to a broader damaged region. In models S5 and S7, the stress distributions of the outer steel tube shown in Fig. 9(a) are relatively uniform, and the primary failure mode in both cases is fracture of the steel tube at the end region. The failure stress is approximately 265 MPa in both cases. As shown in Fig 9(c), the maximum loads of S5 and S7 are 707 kN and 895 kN, respectively, while the corresponding microstrains are 18863 and 19879. These results indicate that, as the confinement effect coefficient increases, the overall stress distribution within the member becomes more uniform, and the crack propagation pattern gradually changes from localized concentration to a more balanced development. Meanwhile, concrete cracks progressively extend toward the chamfered corner regions, suggesting that the outer steel tube may play a certain role in regulating local damage evolution and crack propagation paths.

Overall, the results in Fig 9(a)–9(c) indicate that, as the confinement effect coefficient increases, the failure mode of the core concrete gradually changes from localized tensile cracking to a more uniformly distributed damage pattern. For axially tensioned members, the role of the outer steel tube does not mainly lie in directly enhancing the concrete bearing capacity through lateral confinement, as in compression members, but is more closely associated with strengthening the interfacial cooperation between the steel tube and the concrete, delaying local crack propagation, and improving the overall working performance of the member. When the confinement effect coefficient approaches 1, the member exhibits a more uniform crack evolution pattern, and the final failure is still dominated by fracture of the steel tube. This further demonstrates that, under the conditions considered in this study, the outer steel tube plays the dominant role in resisting tension in axially tensioned members.

#### 3.3.3 Analysis of tensile properties of concrete filled steel tube under different slenderness ratios.

The slenderness ratio is commonly used to calculate the flexibility and stability of structural components. The formula for the slenderness ratio is as follows:


$$\begin{array}{c} \lambda =\frac{\mu l}{i} \end{array}$$ (8)

**Fig 10:**
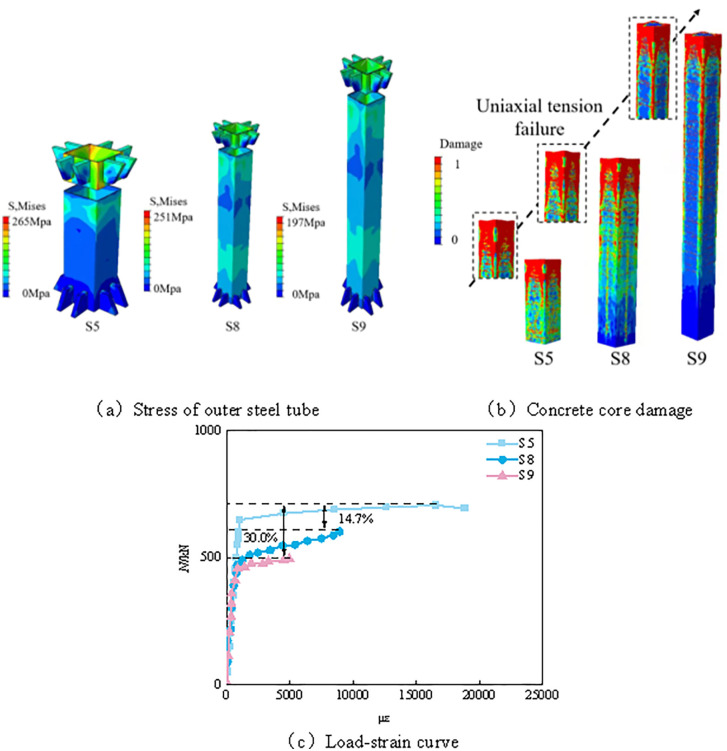
Analysis of tensile properties of concrete filled steel tube under different slenderness ratios. **(a)** Stress of outer steel tube. **(b)** Concrete core damage. **(c)** Load-strain curve.

Where *μ* is the effective length factor, with *μ* = 2 for a member fixed at one end and free at the other end; *l* is the member length; and *i* is the radius of gyration of the specimen. As shown in Fig 10, under different slenderness ratios, both the damage pattern of the core concrete and the load–strain response of the axially tensioned CFST members exhibit certain differences. Overall, with the increase in slenderness ratio, the damage range of the core concrete in the chamfered corner region becomes more extensive; however, failure remains mainly concentrated at the upper end of the member, showing typical tensile failure characteristics, while the other regions are still dominated by transverse cracking. Therefore, the overall failure pattern does not change fundamentally.

As shown by the numerical simulation results in Fig 10(c), the load-carrying capacity and deformation capacity of specimens with different slenderness ratios differ significantly. The maximum load of specimen S5 is approximately 707 kN, corresponding to a maximum microstrain of 18863; the maximum load of S8 is about 603.7 kN, with a maximum microstrain of 675 μm; and the maximum load of S9 further decreases to 495.4 kN, corresponding to a maximum microstrain of 8945. These results indicate that, under the modeling conditions considered in this study, the maximum load of the member generally shows a decreasing trend as the slenderness ratio increases.

From the perspective of deformation response, the variation in slenderness ratio also has a pronounced effect on the ultimate strain. Combined with the load–strain curves in Fig 10(c), it can be seen that, with increasing slenderness ratio, the deformation characteristics of the member before and after reaching the peak load change noticeably, reflecting a variation in the overall load–strain response, which is manifested by a reduction in the equivalent stiffness. In particular, under relatively large slenderness ratios, the deformation development of the member becomes more sensitive, which corresponds well to the observed decline in load-carrying capacity.

In addition, the stress distributions of the outer steel tube shown in Fig 10(a) indicate that the stresses in S5, S8, and S9 are 265 MPa, 251 MPa, and 197 MPa, respectively, showing an overall decreasing trend. This indicates that, as the slenderness ratio increases, the stress level in the outer steel tube gradually decreases, and its contribution to the overall member response correspondingly weakens, thereby affecting the load–strain behavior of the member. Meanwhile, the concrete damage patterns in Fig 10(b) show that increasing the slenderness ratio does not significantly alter the fundamental failure mode of the member, although it enlarges the local damage range to a certain extent. In summary, the results in Fig 10(a)–10(c) indicate that increasing the slenderness ratio reduces the overall load-carrying capacity and renders the deformation response more sensitive, while the basic tensile failure mode remains generally unchanged.

### 3.4 Comparative analysis of tensile strength of different types of concrete filled steel tube and calculated values of each specification

To assess the applicability of tensile bearing capacity calculations for concrete-filled steel tube (CFST) members according to various national and regional codes, this study compares the finite element simulation results with the calculation results from the American, British, European, and Chinese codes. In the calculation formulas of each code, *N*_tu_ represents the design tensile bearing capacity of the CFST member, *A*_s_ represents the cross-sectional area of the steel tube, *f*_y_ represents the yield strength of the steel, *φ*_t_ is the material reduction factor, and *r*_m_ and *r*_m0_ are the material partial safety factors. The yield strength of steel, f_y, in this study is determined based on steel material test results from literature. When *t* = 2.75 mm, *f*_y_ = 298MPa; when *t* = 3.5 mm, *f*_y_ = 311 MPa; and when *t* = 4.5 mm, *f*_y_ = 308 MPa. Based on these parameters, the bearing capacity is calculated using the formulas from different codes.

The American code AISC/ANSI 360−10 uses the following formula to calculate the tensile bearing capacity of the steel tube [44]:


$$\begin{array}{c} N_{tu1\;}=\varphi_{t\;}A_{s}\;f_{y} \end{array}$$ (9)

The material reduction factor is *φ*_t_ = 0.9. The British code BS 5400−2005 adopts [45]:


$$\begin{array}{c} N_{tu2\;}=A_{s}\;\left(\frac{f_{y}\;}{r_{m}}\;\right) \end{array}$$ (10)

The material partial safety factor is *r*_m_ = 1.2. The European code EN 1994-1-1:2004 adopts [46]:


$$\begin{array}{c} N_{tu3\;}=A_{s}\;\left(\frac{f_{y}\;}{r_{m0}}\;\right) \end{array}$$ (11)

The material partial safety factor is *r*_m0_ = 1.0. The Chinese code “Technical Code for Concrete-Filled Steel Tube Structures” GB 50936–2014 and the Fujian Provincial Local Standard DBJ/T 13-51—2003 adopt [47,48]:


$$\begin{array}{c} N_{tu4}\leq 1.1A_{s}f_{y} \end{array}$$ (12)

It can be seen that the first three codes primarily calculate the bearing capacity based on the cross-sectional area of the empty steel tube and the yield strength of the steel, while the Chinese code makes an appropriate adjustment to the bearing capacity on this basis. To quantitatively evaluate the difference between the calculation results of each code and the simulation results, this study uses the relative error index:


$$\begin{array}{c} N_{tu4}\leq 1.1A_{s}f_{y} \end{array}$$ (13)

This section may be divided by subheadings. It should provide a concise and precise description of the experimental results, their interpretation, as well as the experimental conclusions that can be drawn.

As shown in Table 5, the calculation results indicate that all four code provisions are conservative overall, although their error levels differ significantly. The relative error range of the AISC specification is −40.93% to −7.85%, with an average error of −28.05%. For BS 5400, the error range is −45.31% to −14.68%, with an average error of −33.39%, indicating the most pronounced conservatism among the four provisions. The error range of EN 1994 is −34.37% to 2.39%, with an average error of −20.06%. In contrast, the Chinese codes GB 50936–2014 and DBJ/T 13-51—2003 show an error range of −27.80% to 12.63%, with an average error of −12.73%, and their overall absolute errors are the smallest, showing the best agreement with the numerical simulation results. In particular, for most specimens, the calculated values given by the Chinese codes are the closest to the finite element results, indicating a higher level of consistency within the range of specimens considered in this study.

**Table 5:** Comparative analysis of tensile strength of different types of concrete filled steel tube and calculated values of each specification.

Test piece	Value of simulation/kN	AISC/kN	AISC error/%	BS5400/kN	BS5400 error/%	EN1994/kN	EN1994 error/%	GB50936/kN	GB50936 error/%
S1	385.00	286.91	−25.48	265.65	−31.00	318.79	−17.20	350.66	−8.92
S2	452.16	345.91	−23.50	320.29	−29.16	384.35	−15.00	422.78	−6.50
S3	550.76	404.92	−26.48	374.92	−31.93	449.91	−18.31	494.90	−10.14
S4	563.00	378.15	−32.83	350.13	−37.81	420.16	−25.37	462.18	−17.91
S5	707.04	456.52	−35.43	422.70	−40.22	507.24	−28.26	557.97	−21.08
S6	905.52	534.89	−40.93	495.27	−45.31	594.32	−34.37	653.75	−27.80
S7	895.00	576.30	−35.61	533.61	−40.38	640.33	−28.45	704.37	−21.30
S8	603.70	456.52	−24.38	422.70	−29.98	507.24	−15.98	557.97	−7.57
S9	495.40	456.52	−7.85	422.70	−14.68	507.24	2.39	557.97	12.63

The above differences are mainly attributed to the varying extent to which each code considers the interaction between the steel tube and the core concrete. It should be noted that the code formulas compared in this study, including AISC, BS 5400, EN 1994, GB 50936–2014, and DBJ/T 13-51—2003, were established under different application backgrounds and theoretical assumptions. Some of these expressions are essentially simplified approaches developed for empty steel tubes or general composite members under tension, rather than being specifically proposed for square CFST members subjected to axial tension as investigated herein. Therefore, it is understandable that discrepancies exist between the code-based predictions and the numerical simulation results.

From the comparison results, the tensile bearing capacity expressions adopted in the American, British, and European codes are still mainly based on the load-carrying behavior of the steel tube itself, while the consideration of the relevant interaction effects is relatively simplified. As a result, their predictions are generally conservative. By contrast, the Chinese code GB 50936–2014 and the Fujian local standard DBJ/T 13-51—2003 adopt the form of 1.1*A*_s_ *f*_y_ in their formulas, which reflects, to a certain extent, the beneficial effect of concrete infill on the tensile bearing capacity of the steel tube. The corresponding increase is approximately 5%–10%. Within the parameter range of the specimens investigated in this study, this treatment shows closer agreement with the numerical simulation results.

Therefore, within the parameter range of the square CFST members under axial tension considered in this study, the Chinese code predictions are in relatively good agreement with the finite element results and exhibit smaller errors than the other compared code provisions, indicating a certain degree of engineering reference value. Nevertheless, it should also be noted that this conclusion is mainly applicable to the member type, material parameters, and loading conditions involved in this study. Further verification is still required for CFST members under axial tension with other cross-sectional forms, boundary conditions, or broader parameter ranges.

### 3.5 Machine learning analysis

Since the ultimate load and displacement characterize the load-bearing performance and deformation performance of the member, respectively, and their response mechanisms are not completely identical, this study established four machine learning models, namely KNN, Decision Tree, XGBoost, and Random Forest, by taking the ultimate load and displacement as separate prediction targets. The predictive performance of the models was comprehensively evaluated using both the coefficient of determination (R^2^) and absolute error metrics, including the root mean square error (RMSE), mean absolute error (MAE), and mean square error (MSE). Among these indicators, R^2^ reflects the overall goodness of fit, while RMSE, MAE, and MSE provide more intuitive measures of the absolute prediction deviation and error magnitude. Therefore, the combined use of these metrics allows a more comprehensive comparison of the predictive accuracy and stability of different models.

**Fig 11:**
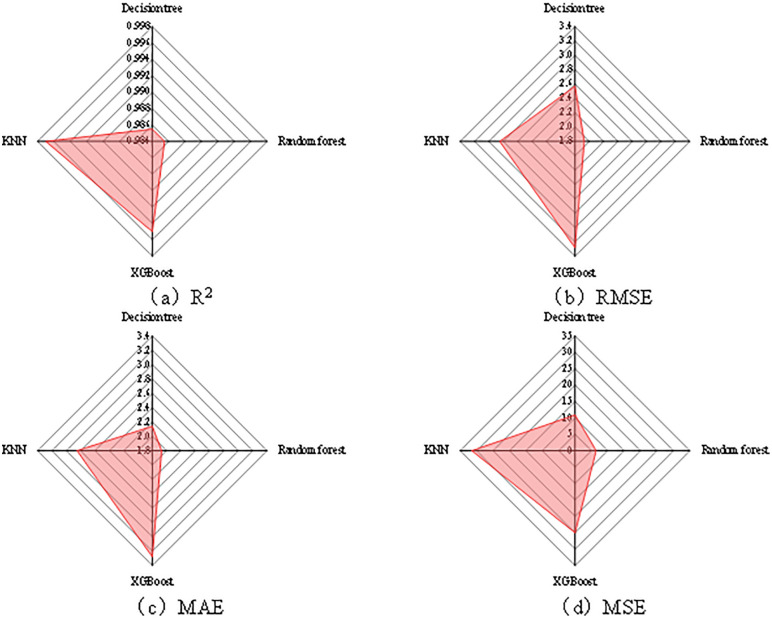
Comparison of prediction performance of different machine learning models for displacement. **(a)** R^2^. **(b)** RMSE. **(c)** MAE. **(d)** MSE.

As shown in Fig 11, for the displacement prediction task, all four models exhibit relatively high goodness of fit, with R^2^ values greater than 0.98. However, when the absolute error measures RMSE, MAE, and MSE are also taken into account, clearer differences in prediction deviation among the models can be identified.Specifically, the Decision Tree model yields an R^2^ of 0.9855, with RMSE, MAE, and MSE values of 2.57, 2.14, and 10.88, respectively; the Random Forest model yields an R^2^ of 0.9855, with RMSE, MAE, and MSE values of 1.93, 1.93, and 6.43, respectively; the XGBoost model yields an R^2^ of 0.995, with RMSE, MAE, and MSE values of 3.28, 3.28, and 25.05, respectively; and the KNN model yields an R^2^ of 0.997, with RMSE, MAE, and MSE values of 2.85, 2.85, and 31.49, respectively. It can be observed that although KNN and XGBoost perform better in terms of the R^2^ metric, their error levels are significantly higher than those of the Random Forest model. In contrast, the Random Forest model achieves the best results in terms of RMSE, MAE, and MSE, indicating its superior error control capability and prediction stability for displacement prediction.

**Fig 12:**
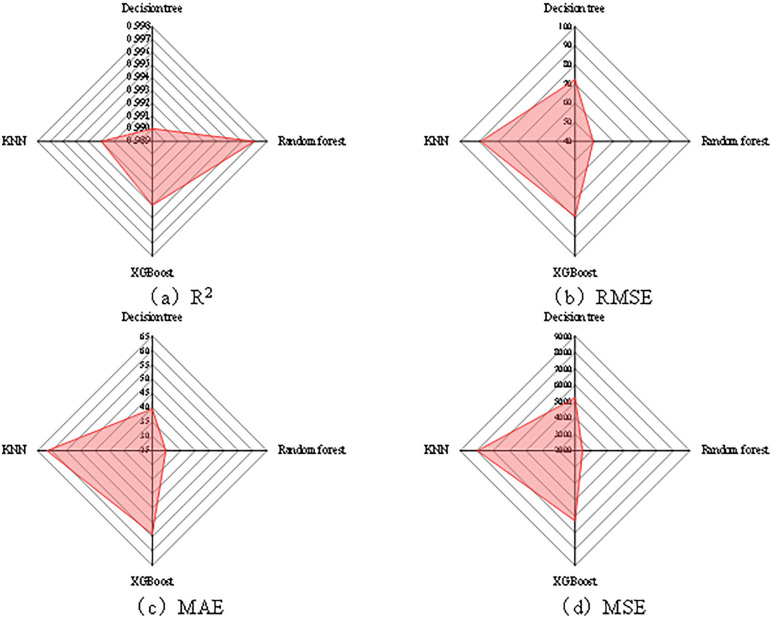
Comparison of load prediction performance of different machine learning models. **(a)** R^2^. **(b)** RMSE. **(c)** MAE. **(d)** MSE.

As shown in Fig 12, for the ultimate load prediction task, all four models also show high predictive accuracy, with R^2^ values greater than 0.99. Meanwhile, the absolute error indicators RMSE, MAE, and MSE provide a more direct basis for comparing the magnitude of prediction errors among different models.Specifically, the Decision Tree model yields an R^2^ of 0.990, with RMSE, MAE, and MSE values of 72.3, 39.6, and 5232.1, respectively; the Random Forest model yields an R^2^ of 0.997, with RMSE, MAE, and MSE values of 49.5, 29.7, and 2459.2, respectively; the XGBoost model yields an R^2^ of 0.994, with RMSE, MAE, and MSE values of 79.2, 54.1, and 6277.5, respectively; and the KNN model yields an R^2^ of 0.993, with RMSE, MAE, and MSE values of 89.2, 61.7, and 7952.7, respectively. A comprehensive comparison shows that the Random Forest model not only achieves the highest R^2^ for ultimate load prediction, but also outperforms the other models in RMSE, MAE, and MSE, thus exhibiting relatively superior overall performance under the evaluation metrics adopted in this study.

Overall, the individual metrics of the different models vary to some extent in the two prediction tasks of displacement and ultimate load, suggesting that the data-mapping relationships corresponding to the load-bearing response and deformation response of the member are not entirely identical. However, based on the comprehensive evaluation results for both target variables, the Random Forest model shows a more balanced performance under the evaluation metrics adopted in this study and was therefore selected as the representative model for subsequent interpretability analysis. For the purpose of maintaining a unified interpretation framework and ensuring consistency in the subsequent feature contribution analysis, the Random Forest model was ultimately selected as the unified model for predicting both the ultimate load and displacement, and SHAP analysis was further conducted. Therefore, greater emphasis is placed in this study on comprehensive error control capability and model stability, rather than selecting the optimal model solely based on a single R^2^ metric.

Although the machine learning models show high predictive performance with R^2^ values greater than 0.99 in both displacement and ultimate load prediction tasks, it is important to note that the testing set used in this study was derived from the augmented data domain, not from independent real samples. Therefore, the reported high R^2^ values primarily indicate strong predictive performance within the expanded data space, which was augmented using the WGAN-GP method. This suggests that the models are effective for interpolation within the parameter range and distribution of the augmented dataset. However, the generalization capability of the models beyond this domain, particularly for extrapolation to new, real-world data, remains uncertain and requires further validation with independent real samples.

To further evaluate the model’s generalization capability on samples that were not involved in the training and data augmentation process, this study adds five completely independent finite element validation samples (see Table 6), forming an independent validation set. Based on the trained Random Forest model, the ultimate load and displacement for this validation set were predicted and compared with the independent finite element results. It should be noted that, although an independent validation set was introduced, the overall sample size remains relatively limited. Therefore, the relatively high R^2^ primarily indicates that the model has strong interpolation prediction capabilities within the parameter range and distribution domain considered in this study. However, for extrapolation predictions that significantly exceed the existing parameter boundaries, the reliability of these predictions still requires further validation using additional experimental data or high-quality finite element samples.

**Table 6:** The prediction results of independent validation set are compared with the finite element results.

Sample No.	Sample literature: *P*/kN	Sample literature: *Δ*/mm	Model prediction: *P*/kN	Model prediction: *Δ*/mm	*P* error/%	*Δ* error/%
G11-220-4-510-235-30	788.54	1.22	738.33	1.17	6.8	4.7
G12-220-5-510-235-30	971.61	1.25	920.09	1.19	5.6	4.9
G13-220-6-510-235-30	1154.17	1.29	1105.53	1.21	4.4	6.9
G14-200-5-510-235-30	879.07	1.20	836.41	1.15	5.1	4.2
G15-240-5-510-235-30	1068.36	1.27	1026.28	1.25	4.1	1.7

**Fig 13:**
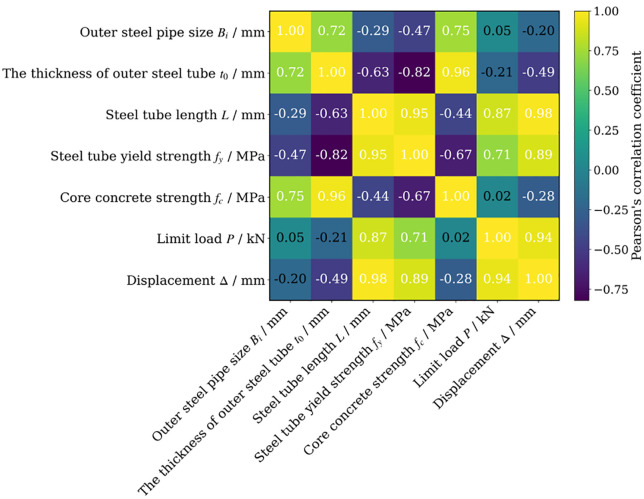
Correlation coefficient matrix.

As shown in Fig 13, first, among the input variables, the outer steel tube size *B*_ᵢ_ is positively correlated with the outer steel tube thickness *t*_0_, indicating that, within the sample set, a larger outer steel tube size is often accompanied by a greater tube thickness. Meanwhile, the outer steel tube thickness *t*_0_ also shows a strong positive correlation with the core concrete strength *f*_c_, suggesting that these two parameters exhibit a certain degree of coordinated variation in the dataset. Relatively speaking, the steel tube length *L* is highly correlated with the steel yield strength *f*_y_, implying that longer members are often associated with higher steel strength. On the other hand, the thickness *t*_0_ and the yield strength *f*_y_ exhibit a strong negative correlation, indicating that some parameters may have a substitutional or balancing relationship, that is, when one performance-related parameter increases, the other does not necessarily increase simultaneously.

From the perspective of the output variables, the ultimate load *P* shows strong positive correlations with both the outer steel tube size *B*ᵢ and the steel yield strength *f*_y_, indicating that these two variables play a significant role in improving the load-carrying capacity. In particular, the relatively strong correlation between steel tube length and ultimate load suggests that the geometric scale has a pronounced influence on the load-bearing response of the member. The displacement *Δ* is also significantly positively correlated with the outer steel tube size *B*_ᵢ_, the steel yield strength *f*_y_, and the ultimate load *P*, indicating that the displacement response is closely related to member size, material strength, and load-bearing level. This further suggests that, as the load-carrying capacity increases, the deformation response of the member also tends to become more pronounced.

In addition, the linear correlation between the core concrete strength and the output responses is relatively weak. Although the steel tube length exhibits a certain degree of correlation with both ultimate load and displacement, its effect still needs to be further examined in conjunction with nonlinear models. Overall, the figure indicates that steel tube length and steel strength are strongly associated with the mechanical response of the member, while a certain degree of collinearity also exists among the input variables. Therefore, in subsequent machine learning modeling, SHAP analysis and feature importance results should be combined to further identify the governing parameters, so as to improve model interpretability and prediction reliability. It should be noted that correlation analysis mainly reflects the degree of linear association between input variables and output responses, whereas SHAP analysis reveals the marginal contribution and direction of effect of variables within the machine learning model. Since machine learning models are capable of capturing nonlinear relationships and interaction effects among variables, it is reasonable that some differences exist between the two methods in terms of feature importance ranking.

**Fig 14:**
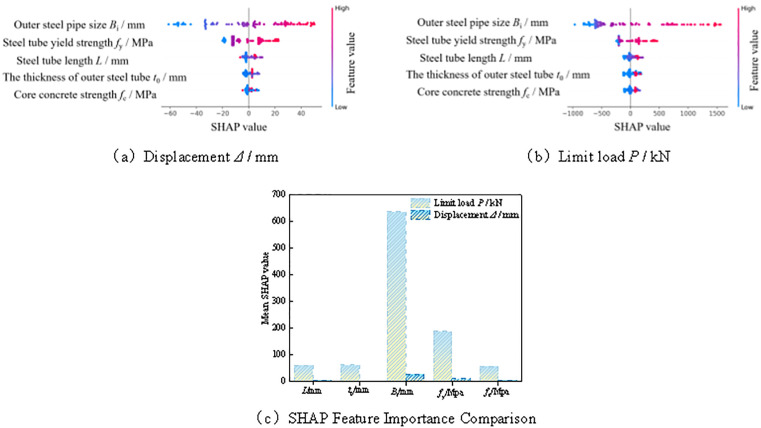
SHAP value analysis. **(a)** Displacement *Δ*/ mm. **(b)** Limit load *P*/ kN. **(c)** SHAP Feature Importance Comparison.

As shown in Fig 14, the extent and direction of influence of different input variables on the displacement *Δ* and ultimate load *P* are markedly different, indicating that although the deformation response and load-bearing response of the member are governed by the same set of parameters, their dominant mechanisms are not entirely identical. In the SHAP plots, the horizontal axis represents the SHAP value; the greater its absolute value, the more significant the contribution of the corresponding variable to the model prediction. The color of the points ranges from blue to red, representing low to high feature values, respectively, which further enables a qualitative assessment of whether the variation of a variable promotes or suppresses the target response. Meanwhile, the mean SHAP values listed in the table quantitatively reflect the overall contribution of each input variable to the model output.

For displacement *Δ*, the outer steel tube size *B*_i_ has the largest mean SHAP value, reaching 27.1, which is significantly higher than those of the other variables. This indicates that *B*_i_ makes the most prominent contribution to displacement prediction and is the most influential input feature in the current Random Forest model. From the SHAP distribution, larger values of *B*_i_ are mostly associated with positive SHAP values, whereas smaller values are mainly associated with negative SHAP values, suggesting that the predicted displacement generally increases with the increase in outer steel tube size. The steel tube yield strength *f*_y_ has the second largest mean SHAP value, equal to 11.8, indicating that it also has a pronounced influence on displacement. By contrast, the mean SHAP values of the steel tube length *L*, core concrete strength *f*_c_, and outer steel tube thickness *t*_0_ are 5.4, 5.2, and 2.9, respectively, implying relatively smaller contributions. This suggests that the direct effects of these variables on displacement are limited and may be more strongly reflected through their coupled interactions with other variables.

For ultimate load *P*, the outer steel tube size *B*_i_ again exhibits the strongest controlling effect, with a mean SHAP value as high as 636.8, far exceeding those of the other features. This indicates that, in the current model, *B*_i_ contributes the most to the prediction of load-bearing capacity. The mean SHAP value of the steel tube yield strength *f*_y_ is 189.5, ranking second only to *B*_i_, which confirms that steel strength has a significant positive contribution to the ultimate load capacity of the member. In comparison, the mean SHAP values of the outer steel tube thickness *t*_0_, steel tube length *L*, and core concrete strength fc are 62.6, 61.8, and 56.9, respectively. Their contributions are relatively close to one another, but still substantially lower than those of *B*_i_ and *f*_y_, indicating that, although they have a certain influence on the prediction results, their contributions in the current model are comparatively less significant.

In the current Random Forest model, the outer steel tube size *B*_i_ and the steel tube yield strength *f*_y_ make relatively large contributions to the prediction of both displacement and ultimate load, among which *B*_i_ is the most influential variable. By comparison, the importance of the outer steel tube thickness *t*_0_, steel tube length *L*, and core concrete strength *f*_c_ is relatively lower. These results indicate that, under the current dataset and model settings, the geometric size parameters of the member and the mechanical properties of steel make more prominent contributions to the prediction results, whereas thickness and concrete strength play more auxiliary roles. This is generally consistent with the previous correlation analysis and machine learning prediction results.

**Fig 15:**
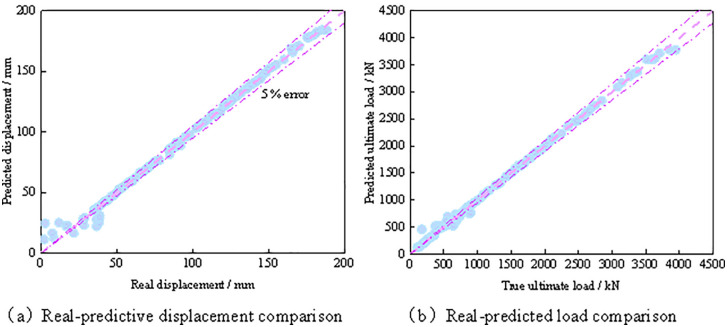
Comparison between predicted and actual values. **(a)** Real-predictive displacement comparison. **(b)** Real-predicted load comparison.

As shown in Fig 15, the predicted values are generally in good agreement with the experimental values, and the relative errors of most samples are controlled within 5%, indicating that the established machine learning model has high prediction accuracy. For the prediction of displacement *Δ*, the predicted results also exhibit a high degree of consistency with the experimental values, with only a small overall level of dispersion, demonstrating that the model is also capable of accurately capturing the deformation response of the member. Overall, most samples fall within the 5% error band, further confirming that the proposed machine learning model possesses high predictive accuracy and good fitting capability.

## 4 Conclusions

1. The established three-dimensional mesoscopic finite element model shows good agreement with the experimental results. The ratios of the experimental values to the simulated values are all less than 0.95, indicating that the model can effectively capture the load–strain response and typical failure characteristics of square concrete-filled steel tube (CFST) members under axial tension.
2. The parametric analysis shows that the ultimate bearing capacity of the member increases with the increase in section size. With the increase in the confinement effect coefficient, the interfacial cooperative action is enhanced and the damage distribution becomes more uniform. When the slenderness ratio increases from 8 to 20, the maximum load of the member generally shows a decreasing trend.
3. The code comparison indicates that, within the parameter range considered in this study, the predictions given by the Chinese code are closer to the finite element results. The average predicted value is 2064.24 kN, with an error of approximately 4.57%, showing better agreement than the corresponding European and American code provisions. However, it is important to note that the conclusions drawn from this comparison are based on a limited parameter range, specifically square CFST members under axial tension. The applicability of these results to other types of CFST members or different loading conditions remains uncertain and requires further research to validate the broader applicability of the findings.
4. Based on 16 original samples and the WGAN-GP-augmented dataset, prediction models were developed, among which the Random Forest model showed relatively better overall performance. On the test set of the augmented samples, the coefficients of determination (R^2^) for ultimate load and displacement prediction reached 0.93 and 0.98, respectively, providing a useful reference for the rapid assessment of member response.

## Supporting information

S1 File Fig 8.opju. Origin project file for the tensile analysis of concrete-filled square steel tube members with different cross-sectional sizes. The file contains the stress distribution of the outer steel tube, damage distribution of the concrete core, and load–strain curves for the corresponding numerical models.
(OPJU)

S2 File Fig 9.opju. Origin project file for the tensile analysis of concrete-filled square steel tube members under different confinement effect coefficients. The file includes the stress distribution of the outer steel tube, concrete core damage patterns, and load–strain curves used to evaluate the influence of confinement on tensile behavior.
(OPJU)

S3 File Fig 10.opju. Origin project file for the tensile analysis of concrete-filled square steel tube members with different slenderness ratios. The file contains the stress distribution of the outer steel tube, damage distribution of the concrete core, and load–strain curves showing the influence of slenderness ratio on axial tensile performance.
(OPJU)

S4 File Fig 12.opju. Origin project file for the comparison of load prediction performance among different machine learning models. The file presents the evaluation results of KNN, Decision Tree, XGBoost, and Random Forest models using R^2^, RMSE, MAE, and MSE.
(OPJU)

S5 File Fig 13.opju. Origin project file for the correlation coefficient matrix of the input and output variables. The file shows the relationships among outer steel tube size, tube thickness, member length, steel yield strength, concrete strength, ultimate load, and displacement.
(OPJU)

S6 File Fig 14.opju. Origin project file for the SHAP value analysis of the Random Forest model. The file presents the feature contributions of the input variables to the predictions of displacement and ultimate load, together with the feature importance comparison.
(OPJU)

S7 File Fig 15.opju. Origin project file for the comparison between predicted and actual values. The file shows the prediction accuracy of the machine learning model for displacement and ultimate load, including the corresponding error distribution.
(OPJU)

S8 File WGANGP.txt. Source code file for the WGAN-GP-based data augmentation procedure. The file contains the implementation used to generate augmented samples from the original small-sample dataset for subsequent machine learning prediction of ultimate load and displacement.
(TXT)
